# Management of Septic Arthritis of the Temporomandibular Joint in Dogs

**DOI:** 10.3389/fvets.2021.648766

**Published:** 2021-03-29

**Authors:** Boaz Arzi, Natalia Vapniarsky, Amy Fulton, Frank J. M. Verstraete

**Affiliations:** ^1^Department of Surgical and Radiological Sciences, School of Veterinary Medicine, University of California, Davis, Davis, CA, United States; ^2^Department of Pathology, Microbiology and Immunology School of Veterinary Medicine, University of California, Davis, Davis, CA, United States; ^3^Aggie Animal Dental Center, Mill Valley, CA, United States

**Keywords:** computed tomography, septic, arthritis, bacteria, temporomandibular joint

## Abstract

Septic arthritis of the temporomandibular joint (TMJ) in dogs and other mammals is a rare condition. It is typically associated with notable pain, swelling, and difficulty in opening the mouth. Unlike degenerative TMJ disease, septic arthritis requires urgent intervention. The etiology of the condition may include penetrating trauma, an extension of local infection, such as otitis media, or the hematogenous spread of a pathogen. However, the precise cause may not always be identified. Diagnostic imaging with Computed Tomography (CT), cone-beam CT (CBCT), and/or Magnetic Resonance Imaging (MRI) are helpful for honing the definitive diagnosis and formulating a treatment plan. Subsequently, exploratory surgery may be required to obtain samples for culture and sensitivity and histology and to lavage the joint. In this “methods” article, we provide a detailed description of our approach to diagnosis and management of septic TMJ arthritis in four dogs.

## Introduction

Septic arthritis of the temporomandibular joint (TMJ) is a rare condition that occurs by seeding pathogens, typically bacteria, by hematogenous route or by direct spread into the joint (1–6). Unlike osteoarthritis of the joint that is considered “low inflammatory” arthritis, septic TMJ arthritis is considered a “high-inflammatory” arthritis and exhibits distinct biological and clinical behavior (6, 7). The development of TMJ septic arthritis is dependent on various factors, such as the pathogen (i.e., bacteria, fungi, or parasite) and its ability to colonize the joint and host-related factors (6).

Affected individuals typically exhibit periauricular pain, pain when opening and closing the mouth, swelling, redness, and occasionally purulent discharge (6, 8–10). Diagnosis of TMJ septic arthritis requires a physical examination and advanced diagnostic imaging, namely CT, cone-beam CT (CBCT), and, occasionally, MRI. Importantly, laboratory analysis of joint fluid obtained by arthrocentesis, or exploratory arthrotomy may be required to optimize the treatment of TMJ septic arthritis (4, 11–13). Although no gold standard exists in the management of TMJ septic arthritis, initiating therapy early, preferably based on precise information obtained from diagnostic imaging, joint decompression, and sampling (i.e., culture and sensitivity), can optimize patient outcomes (7, 14–17).

Although the management of TMJ septic arthritis has been described in human, in many reports, and in animals, in some reports, to the knowledge of the authors, a detailed description of the management of this condition in dogs has not been described in the English peer-reviewed literature in the past 10 years (1, 9, 12, 18, 19). Therefore, we sought to report our experience in the diagnosis and management of TMJ septic arthritis and report the outcome in four dogs.

### Diagnostic Techniques

Dogs that were presented with pain when opening the mouth, swelling of the TMJ area, or pain of unknown origin were screened by conventional CT (HiSpeed FX/i or LightSpeed16, GE Healthcare, Waukesha, WI) and/or CBCT (NewTom 5G CBCT Scanner, NewTom, Verona, Italy or VetCAT, Xoran Technologies, Michigan, Unites States) scans at their initial visit. In most cases, contrast CT studies were acquired after intravenous administration of iopamidol (Isovue 370, Bracco Diagnostics, Monroe Township, NJ, USA).

Images from MRI were acquired as needed using a 1.5T MRI system (GE Healthcare Signa, Waukesha, WI). MRI and conventional CT images were viewed using eFilmWorkstation® 3.3 (Merge Healthcare, Hartland, WI, USA). All CBCT DICOM files were viewed using a specialized software (*In vivo* 5 or *In vivo* 6, Anatomage, San Jose, CA, USA). Each case was evaluated on medical-grade monitors (ASUS PB278Q 27-inch, ASUSTeK Computer Inc., Taipei, Taiwan).

### Surgical Technique

**Figure 1:**
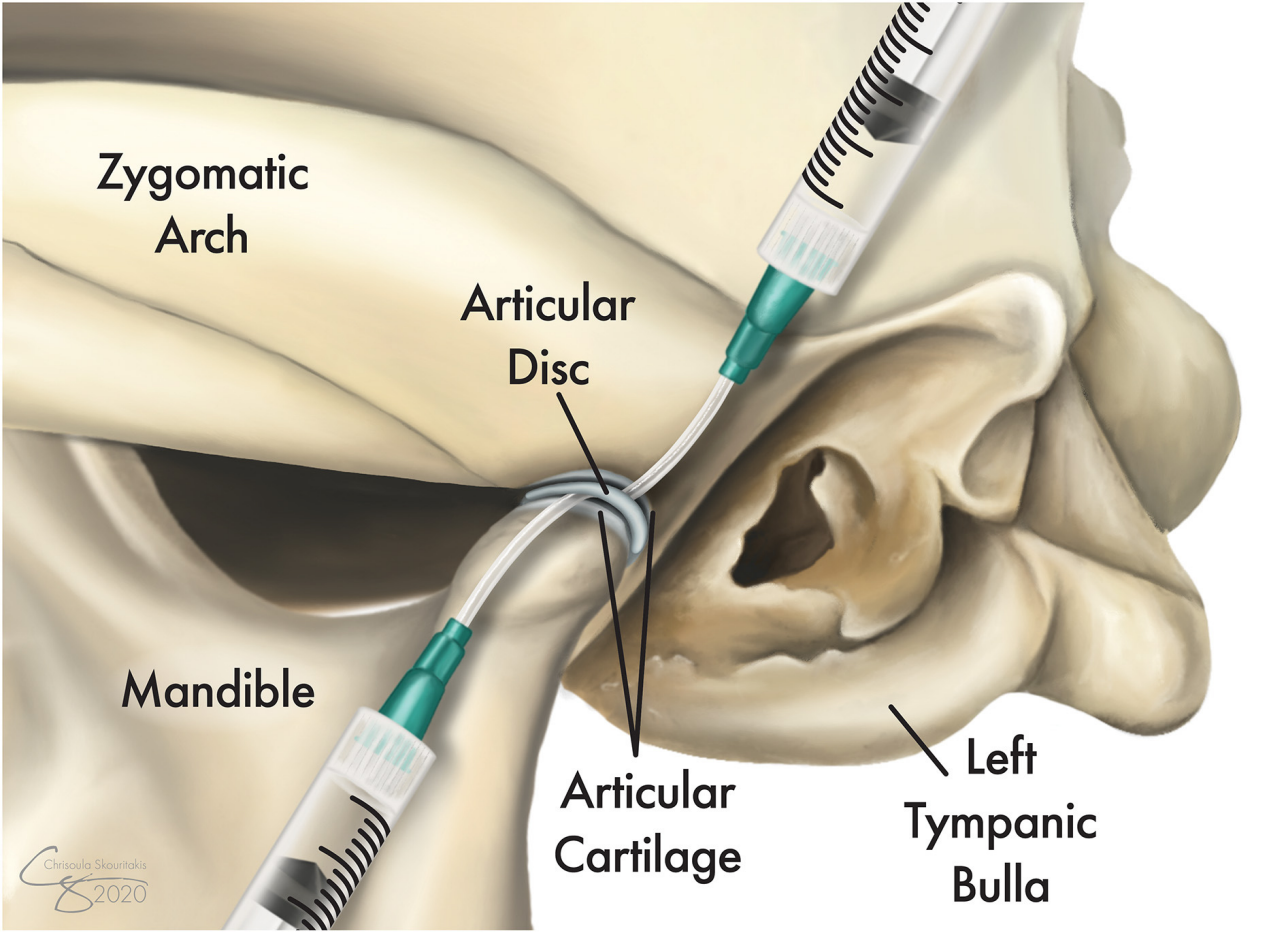
Illustration of the topographic bone and cartilage anatomy of the left temporomandibular joint (TMJ) and its proximity to the tympanic bulla. Following exposure of the lateral aspect of the joint, the TMJ disc is identified and released from its dorsal and ventral attachments to allow full access to both the dorsal and ventral compartments. Tissue samples were obtained from the joint capsule and other grossly affected tissues and were submitted for culture and sensitivity testing as well as for histopathological evaluations. A sterile IV catheter (18G or larger) is inserted into each of the joint compartments and the joint is copiously irrigated with 0.9% sterile saline.

In order to obtain samples for culture and sensitivity testing and for histopathology, a surgical approach to the joint was performed. Furthermore, surgical access to the dorsal and ventral compartments of the TMJ was performed to allow for copious lavage and reduction of bacterial load and gross evaluation of the integrity of the TMJ tissues (Figure 1). The affected side of the head was clipped and aseptically prepared for surgery. The dogs were placed in lateral recumbency with the head and cervical region slightly elevated.

A lateral approach to the TMJ was performed as described in an earlier study (20, 21). A full-thickness skin incision along the caudoventral aspect of the zygomatic arch was performed. Blunt dissection through the platysma muscles and the zygomatic and sphincter colli muscles was performed to expose the masseter muscles. The auriculopalpebral and buccal branches of the facial nerve, as well as the parotid gland and duct, were avoided. The masseter muscular attachment at the ventral aspect of the caudal zygoma was elevated by blunt and sharp dissection using a periosteal elevator. Once the lateral aspect of the TMJ capsule and lateral ligament were identified, a careful horizontal incision using a #15 scalpel blade was performed to expose the joint. Then, the TMJ disc was identified and released from its dorsal and ventral attachments to allow full access to both the dorsal and ventral compartments. If foreign material was identified, it was removed. At this juncture, tissue samples were obtained from the joint capsule and from other grossly affected tissues and submitted for culture and sensitivity testing as well as for histopathological evaluations. Then, antibiotic therapy (ampicillin 20 mg/kg) was administered systemically. After the collection of samples, a sterile IV catheter (18G or larger) was inserted into each of the joint compartments and the joint was copiously irrigated with 0.9% sterile saline (Figure 1). Debridement of soft tissues and bones was performed as needed to remove gross debris. Finally, a routine three-layer closure of the surgical site was performed without drain placement.

## Case Reports

### Case 1

**Figure 2:**
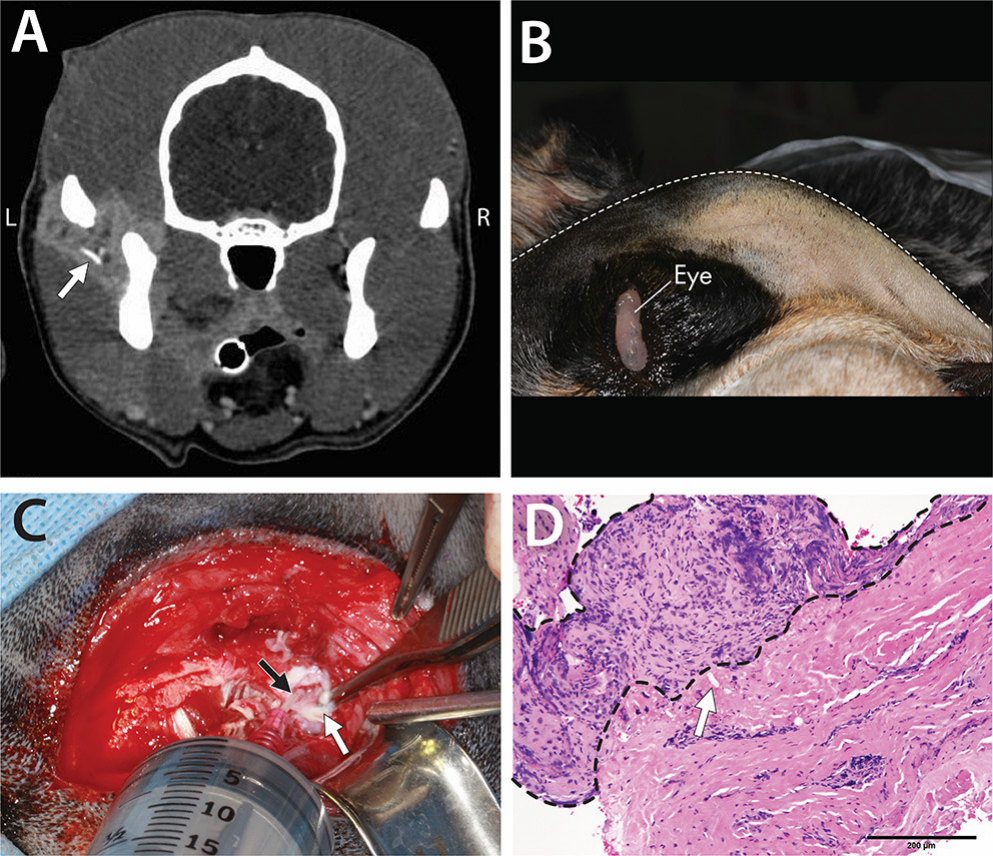
A 5-year-old dog affected by septic arthritis of the left TMJ described in Case 1. **(A)** Contrast-enhanced CT demonstrated inflamed soft tissues as well as a foreign body (arrow) surrounding the TMJ. **(B)** Image obtained with the dog in the right lateral recumbency. Note the substantial swelling on the left side of the face. **(C)** The exposed lateral aspect of the left TMJ with the disc (white arrow) retracted ventrally to allow the 18G catheter (black arrow) to enter the dorsal compartment of the joint for irrigation. **(D)** Histopathological findings demonstrated chronic suppurative inflammation with profound hyperplasia of the joint synovium and extensive fibrosis. Also noted is a foreign body embedded in the tissues (white arrow) [H&E staining and Bar = 200 μm].

A 5-year-old, intact, male Australian cattle dog weighing 23.2 kg was presented for evaluation of oral pain and swelling in the left caudal maxillary and zygomatic regions, which was noticed ~1 month earlier. The dog had a history of a stick injury in the mouth about 1 year earlier. The owner reported that removing the stick, which was followed by some bleeding, self-resolved the issue. At that time, the owner did not seek further medical attention for the dog. Three weeks before the presentation, the referring veterinarian evaluated the dog and obtained pre- and post-contrast CT images. These scans revealed a region of cellulitis and myositis in the proximity of the left TMJ, osteophytes on the medial aspect of the left condylar process, narrowing of the joint space, and periosteal reaction at the lateral side of the left condylar process. Notably, a foreign body was identified lateral to the TMJ (Figure 2A).

An oral and maxillofacial examination revealed swelling on the left maxilla up to the base of the ear, with the apex of the swelling over the TMJ (Figure 2B). The dog exhibited substantial pain in its attempt to open the mouth and was reluctant to allow a fully awake oral examination. Complete blood count and urinalysis were within the normal limits, and the serum biochemistry analysis revealed very mild hyperproteinemia and hyperglobulinemia.

A surgical approach to the TMJ was performed as described above. Upon approaching the TMJ, purulent material was found in the TMJ, and the periarticular area included two small foreign bodies that were grossly suspected to be plant materials. Incisional biopsies of the joint capsule and surrounding tissues were obtained (i.e., intracapsular and extracapsular) and submitted for culture and sensitivity testing and for histopathology. Following copious lavage (Figure 2C), closure was performed routinely. The dog was discharged with the following medications: amoxicillin/clavulanic acid (20 mg/kg, q 12 h for 14 days), enrofloxacin (10 mg/kg, q 24 h for 12 days), tramadol (3 mg/kg q 8–12 h for 8 days), and carprofen (2.2 mg/kg q 12 h for 14 days). The owner was informed that the medical therapy might need to be modified based on the culture and sensitivity results.

Histopathological evaluation revealed severe, chronic, multifocal cellulitis, and steatitis with extensive fibrosis. In addition, small fragments of foreign material were noted (Figure 2D). Culture and sensitivity analysis reported a large number of *Pasteurella canis*, a moderate number of *Streptococcus viridans*, and a small number of *Corynebacterium* species. Anaerobic culture identified mixed growth of beta-lactamase negative *Porphyromonas* species, *Propionibacterium* species, *Bacterioides* species, and *Fusobacterium* species. Given the culture and sensitivity results and after a consultation with a medical pharmacologist, administration of amoxicillin/clavulanic acid continued for 4 additional weeks, and metronidazole at a dose of 21 mg/kg q 12 h for 4 weeks was added. At the 2-week follow-up, the dog was doing well, and the swelling had resolved. A phone interview with the owner revealed that the dog was free of clinical symptoms for the next 7.5 years with no sign of recurrence.

### Case 2

A 7-year-old male, neutered bullmastiff dog, weighing 65.5 kg, was presented for evaluation of right-sided severe masticatory muscle atrophy and pain. The dog was diagnosed with atrophy of the masticatory muscles on the right side and ear infection ~2 months earlier by the referring veterinarian and was treated with topical otic medications to resolve the infection (Posatex®, Merck Animal Health). Antibody testing for masticatory muscle myositis was negative. One month later, the dog exhibited an increase in pain on the right side of the face, was hesitant to eat, and was administered amoxicillin (1,500 mg q 8 h) for an unknown duration. The owner also reported a decrease in the energy level but reported that the dog was still exhibiting a good appetite.

**Figure 3:**
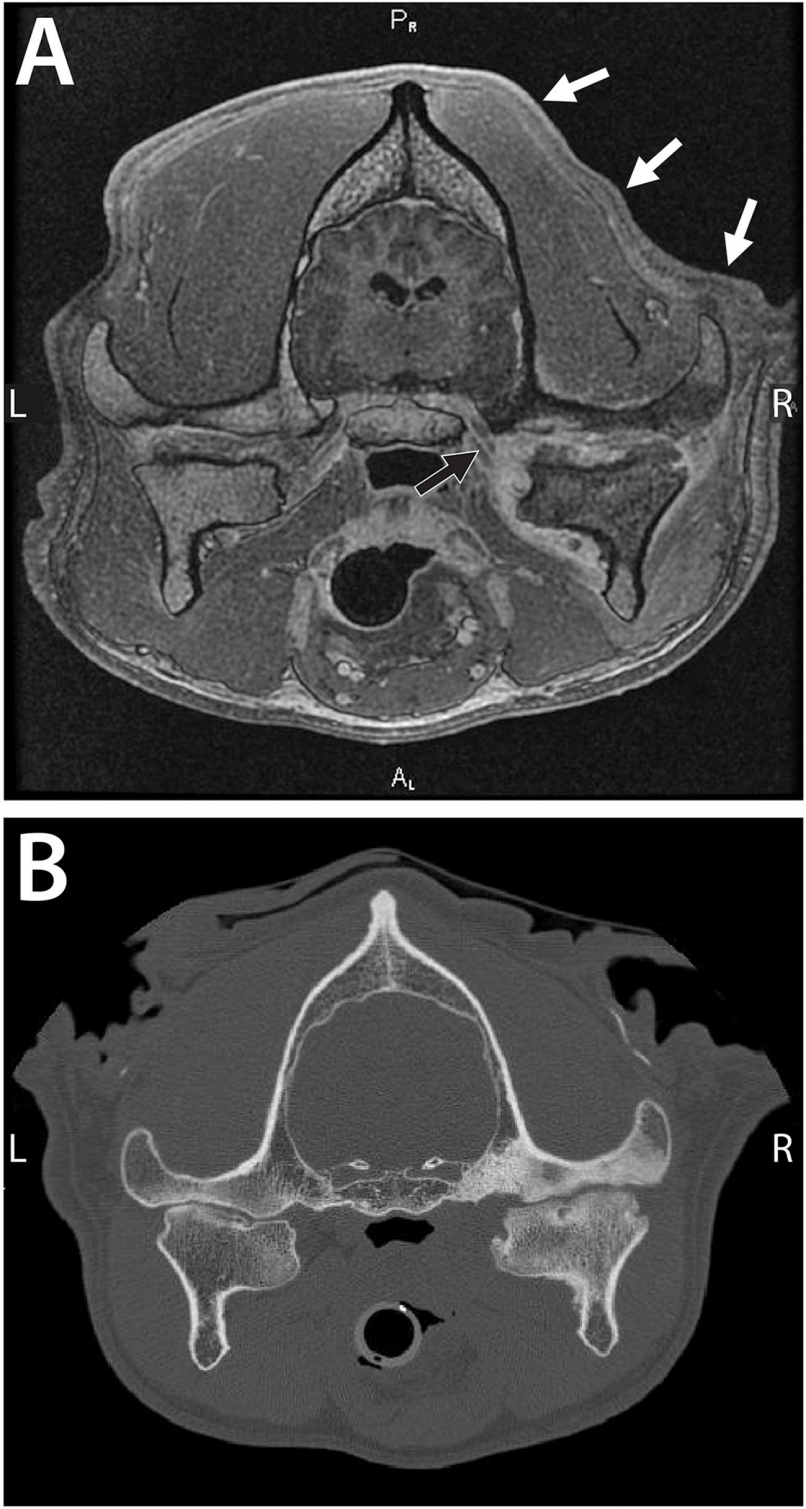
A 7-year-old dog with severe and chronic septic arthritis of the right TMJ described in Case 2. **(A)** Images from MRI demonstrating severe erosive septic arthritis of the right TMJ with concurrent osteomyelitis including involvement of the mandibular branch of the cranial nerve V (black arrow) and associated neuritis. Note the secondary right-sided atrophy of the masticatory muscles and the temporal muscles, in particular (white arrows). **(B)** A conventional CT image demonstrating severe bone sclerosis of the condylar process, mandibular head, and mandibular fossa as well as irregular joint space. In addition, lateral and medial osteophytes were noted as well as a periosteal reaction at the medial aspect of the condylar process and subchondral bone cyst at the mandibular head. Note that the contralateral TMJ exhibits features consistent with advanced degenerative changes (Cissell et al., Diagnostic imaging in oral and maxillofacial surgery. In: Verstraete FJM, Lommer MJ, Arzi B, Editors. Oraland Maxillofacial Surgery in Dogs and Cats, 2nd ed St. Louis, MO: Elsevier; 2020. p. 56-64. Reproduced with permission, copyright Elsevier).

Neurologic examination revealed right-sided atrophy of the temporal and masseter muscles, right-sided facial drooping, and pain on palpation of the right TMJ region. In addition, there were proprioceptive deficits in the pelvic limbs. Complete blood count and serum biochemistry were within the normal limits. Standard thoracic radiographic series were obtained and were within the normal limits; however, an abdominal ultrasound revealed a mural mass at the gastric fundus with mineralization. Standard MRI sequences of the head were obtained followed by a conventional CT scan as described earlier. Severe erosive septic arthritis of the right TMJ with concurrent osteomyelitis was diagnosed, including involvement of the mandibular branch of the trigeminal nerve and associated neuritis (Figure 3). Other changes of the affected joint included a subchondral bone cyst, periosteal reaction at the right condylar process, and osteophytes. Secondary right-sided atrophy, which was likely neurogenic, was also observed. In addition, degenerative changes, such as irregular joint space and joint surfaces and subchondral bone sclerosis, at the left TMJ were also apparent. Cerebrospinal fluid was obtained, and it revealed mild mononuclear reactivity and possible marginal suppurative inflammation. No infectious organisms or neoplastic cells were observed. The owner declined the exploratory approach to the TMJ to obtain samples and an empirical therapy was initiated which included amoxicillin/clavulanic acid (17 mg/kg, q 12 h for 8 weeks), enrofloxacin (10.5 mg/kg, q 24 h for 8 weeks), tramadol (3 mg/kg q 8–12 h), and meloxicam (0.11 m g/kg q 24 h). The dog was lost to short-term follow-up and was presented again 1 year later to emergency service for evaluation of a mass on the chest and for limb edema. The owner reported that the dog exhibited progressive inability to open the mouth since the diagnosis of septic arthritis 1 year earlier. At that time, the dog was diagnosed with undifferentiated round cell neoplasia with local lymphadenomegaly and pitting edema at the right forelimb and was humanely euthanized as per the request of the owner.

### Case 3

An ~3-year-old female, spayed mixed breed dog, weighing 18.5 kg, was presented for a draining tract in the left TMJ area. The draining tract was associated with extensive scar tissue, reduced mandibular range of motion, progressive oral pain, and frequent head-shaking. The dog was adopted 4 months before presentation after being rescued. Since the adoption, the dog was treated for the draining tract with multiple antibiotics with no apparent resolution of the condition.

**Figure 4:**
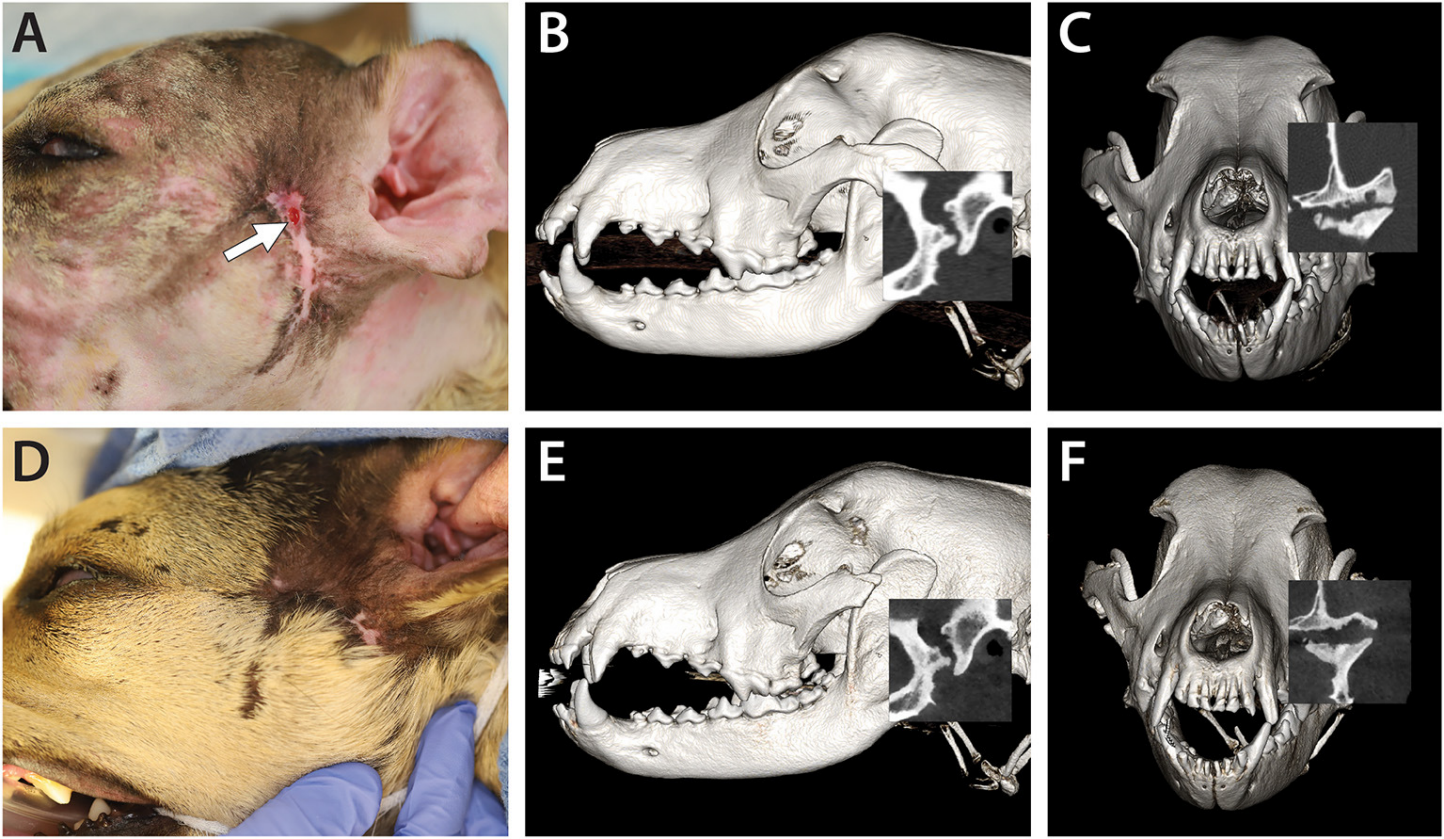
A 3-year-old dog affected by septic arthritis of the left TMJ is described in Case 3. **(A)** Preoperative image demonstrating the extensive scar and the presence of a draining tract in the area of the TMJ (white arrow). **(B)** A lateral three-dimensional image and **(C)** transverse preoperative CBCT image demonstrating severe destruction of the mandibular head and the condylar process as well as erosive destruction of the articular surface of the mandibular fossa. **(D)** A 4-month follow-up image demonstrating the resolution of the draining tract. **(E)** A lateral three-dimensional image and **(F)** a transverse CBCT image at the 4-month follow-up demonstrating static to slightly improved features of the left TMJ.

Oral and maxillofacial examination revealed a moderately atrophied temporal and masseter musculature, substantial scar tissue, and contraction of the skin on the left side of the face. In addition, a draining tract with hemorrhagic purulent discharge at the left TMJ area was noted (Figure 4). Multiple tooth fractures were also noted as well as mild generalized dental plaque and calculus accumulation. It was estimated that mouth opening was ~75% of normal opening on both an awake examination and an examination under general anesthesia. Complete blood count, serum biochemistry, and urinalysis were within the normal limits. Ultrasound of the left TMJ area revealed potential osteomyelitis of the zygomatic arch with suspected sequestrum and regional cellulitis with possible TMJ involvement.

Pre- and post-contrast CT images of the skull were obtained as described earlier. Severe chronic septic arthritis of the left TMJ, characterized by full destruction of the articular surfaces as well as osteomyelitis of the zygoma and sequestrum formation, was noted. In addition, secondary cellulitis, abscessation, and draining tract (Figure 4), as well as enlarged, likely reactive, left mandibular and medial retropharyngeal lymph nodes and chronic otitis at the left ear, were noted.

A lateral approach to the left TMJ was performed as described earlier. In addition to the procedure described above, zygomectomy of the affected bone was performed with piezosurgery (Piezotome® Cube, Acteon, Mérignac, France) from caudal to the orbital process (and just rostral to the lytic bone defect) and to the rostral aspect of the mandibular fossa (not penetrating the joint). Once the infected and grossly necrotic zygomatic arch was removed, the approach to the TMJ was continued. The removed tissues were submitted for culture and sensitivity testing and histopathology. The dog was discharged with the following medications: fentanyl transdermal patch (50 mcg/h), clindamycin (23 mg/kg, q 12 h until sensitivity results were available), gabapentin (10–20 mg/kg q 8–12 h), and carprofen (2.2 mg/kg q 12 h for 14 days). The owner was informed that the medical therapy might need to be modified based on the culture and sensitivity results.

**Figure 5:**
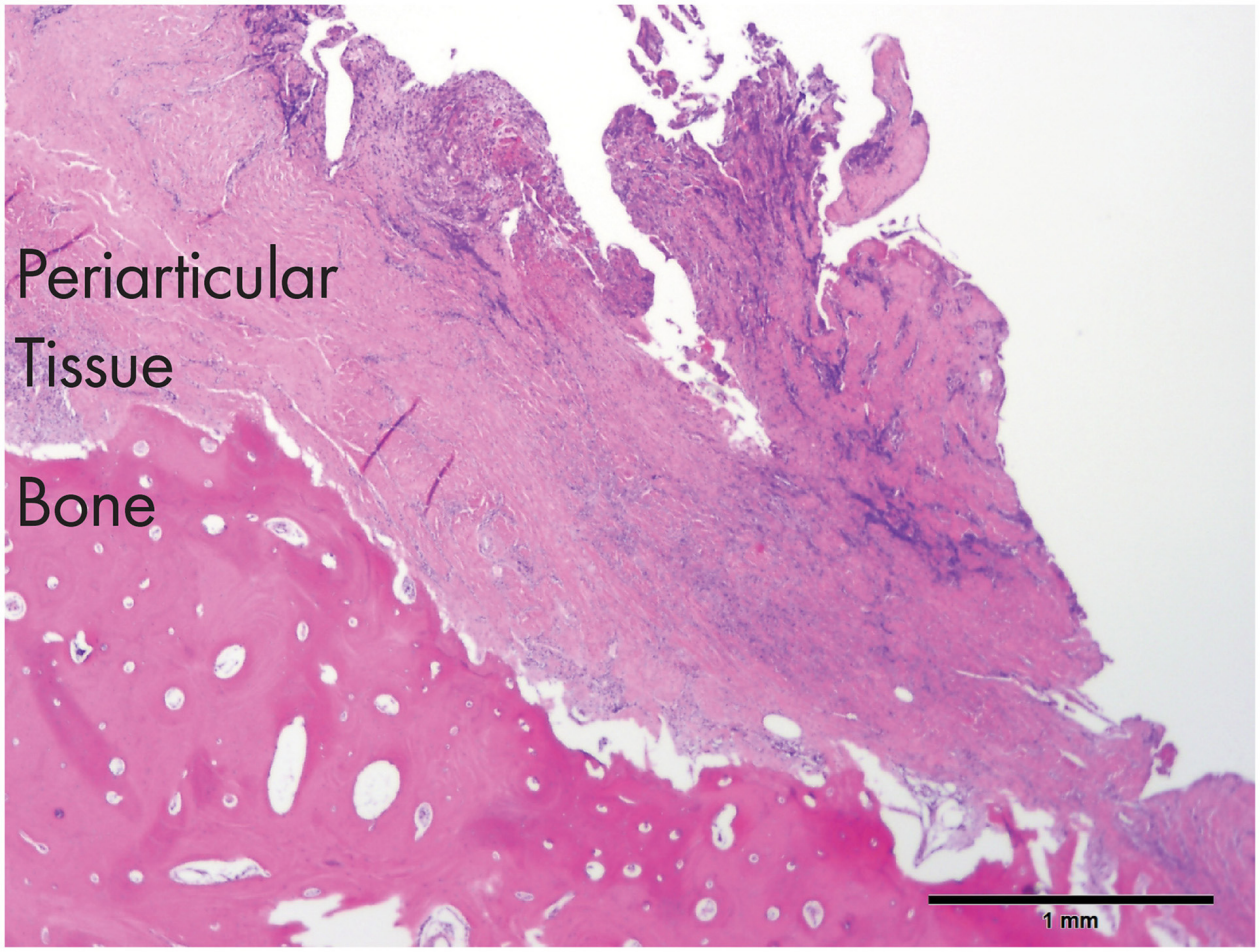
An histological section of the tissues obtained from dog 3. Note the severely inflamed periarticular tissues and the necrotic bone (zygomatic arch) in the proximity of the affected TMJ [H&E staining and Bar = 1 mm].

Histopathological evaluation revealed lymphoplasmacytic, histiocytic, neutrophilic fasciitis, osteomyelitis, and osteonecrosis (Figure 5). Culture and sensitivity analysis yielded a small number of *P. canis*. Anaerobic culture identified a moderate number of mixed growths, including beta-lactamase negative *Bacterioides/prevotella species*, and a small number of *Peptostreptococcus anaerobius* species. Given the culture and sensitivity results, and in consultation with a medical pharmacologist, administration of clindamycin was discontinued, and administration of amoxicillin/clavulanic acid was started for 6 weeks (14 mg/kg, q 12 h), along with metronidazole at a dose of 14 mg/kg q 12 h for 6 weeks. The dog was evaluated at 6 days and 3 weeks postoperatively, and was found to have substantial improvement and resolution of the draining tract. No pain was noted on palpation and the dog was noted yawning multiple times without perceived pain.

An oral and maxillofacial examination was performed at the 4-month recheck appointment, and CBCT images of the skull were obtained (Figures 4D–F). The dog exhibited complete resolution of clinical signs, with no recurrence of the draining tract, and increased mouth opening as visually compared to pretreatment (gape angle 49.6 degrees, interincisive measurement 75 mm). CBCT demonstrated static to slightly improved articular surfaces of the left TMJ with persistent irregular articular margins of both the mandibular head of the condylar process and the mandibular fossa.

### Case 4

An 11-year-old, male, neutered pug, weighing 8.7 kg, was presented for profound pain when opening the mouth. The dog has had, over a year, history of chronic bilateral middle ear disease treated with two ventral bulla osteotomy surgeries. Despite surgery and medical management, the dog continued to experience profound pain. The dog also had concurrent atypical Addison's disease, moderate to severe periodontal disease, and brachycephalic airway syndrome. The dog was receiving a daily dose of prednisone for the management of Addison's disease.

Oral and maxillofacial examination revealed that the dog experienced substantial pain on its attempt to open the mouth which had developed and worsened over the last year and a half. The dog had previously been seen for periodontitis and had had multiple extractions. In the interim between the previous extractions and the current presentation, the dog had had several ear infections that had been difficult to control and had been treated by a specialty group combining veterinary dermatology, surgery, and neurology, given the myriad needs of the dog. After a first bulla osteotomy, the dog developed further ear infection on the right side, and a second bulla osteotomy found a cholesteatoma on the right side. After the second surgery, the dog developed worsening signs of pain, difficulty in opening the mouth, and eating. On oral examination, apart from the previously extracted teeth and moderate dental calculus, no obvious oral abnormalities were detected. The dog was reluctant to allow an open-mouth examination or palpation of the ears and TMJ. Complete blood count and urinalysis were within the normal limits and the serum biochemistry analysis revealed very mild hyperproteinemia and hyperglobulinemia.

**Figure 6:**
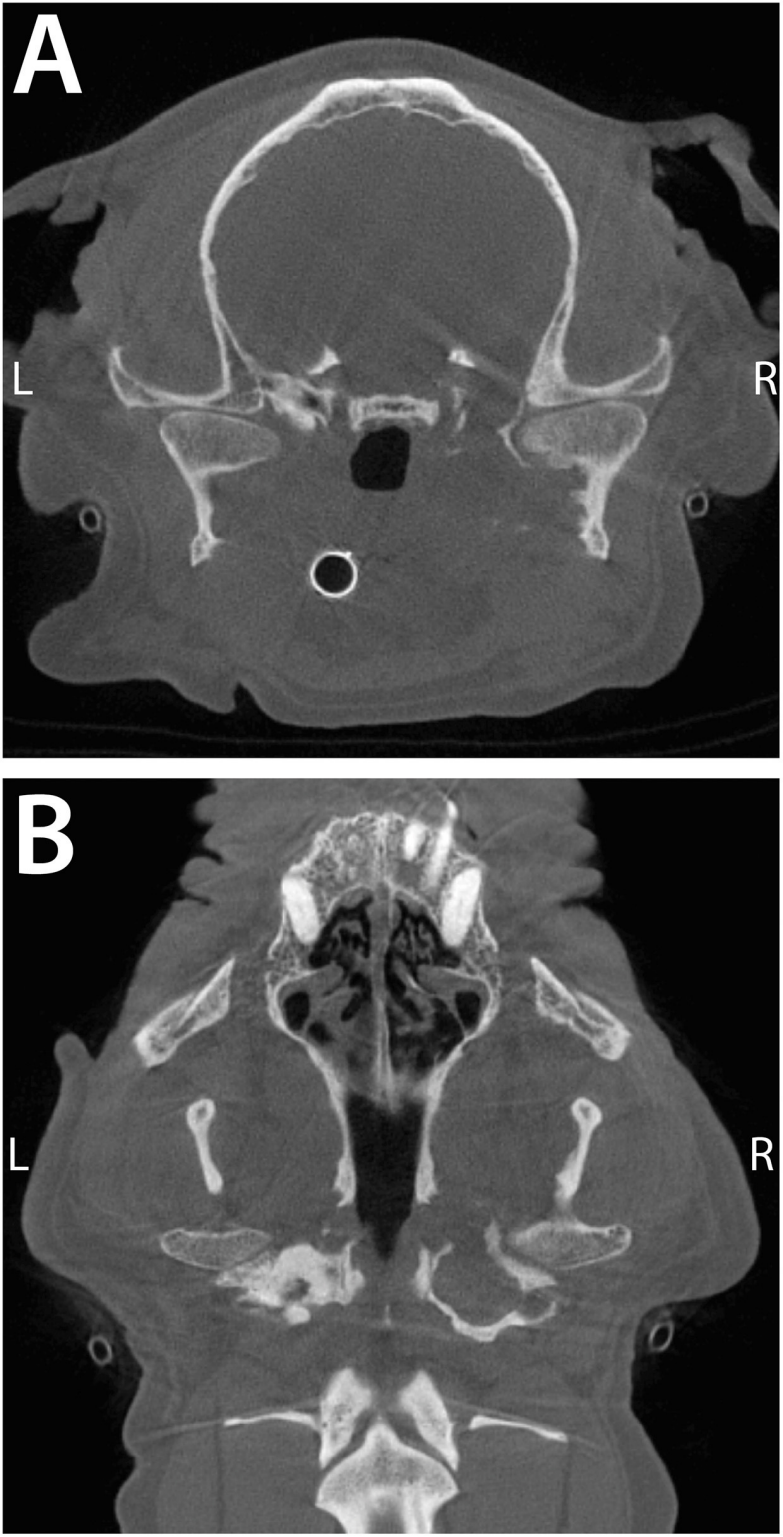
Transverse **(A)** and dorsal **(B)** CBCT images of a 11-year-old, dog described in Case 4. Note the severe periosteal reaction at the medial aspect of the right condylar process and the engulfment of the medial aspect of the TMJ by the deformed bulla. The intimate proximity of the middle ear to the TMJ provides a readily available source for infection to the TMJ.

Cone-beam CT images of the skull were obtained. Severe erosive arthritis of the right TMJ was observed, which was characterized by a pronounced periosteal reaction of the condylar process, osteophytes present on the medial aspect of the joint, and subchondral bone cyst at the lateral aspect of the condylar process. There was also increased density of the bone of the condylar process as compared to the left side, suggesting a septic process. Importantly, the bulla on the right side was deformed, with bony expansion, and it engulfed the medial aspect of the condylar process (Figure 6). A surgical approach to the TMJ area was performed as described earlier. Fluid and samples were obtained from the joint capsule and the surrounding tissues and were submitted for culture and sensitivity testing. An enlarged Lymph node in the proximity of the joint was removed and submitted for histopathological analysis.

The dog was discharged with the following medications: amoxicillin/clavulanic acid (20 mg/kg, q 12 h for 14 days), enrofloxacin (10 mg/kg, q 24 h for 12 days), tramadol (3 mg/kg q 8–12 h for 8 days), and carprofen (2.2 mg/kg q 12 h for 14 days). The owner was informed that medical therapy may need to be modified based on the culture and sensitivity results.

Histopathological evaluation of the lymph node revealed a reactive process with no evidence of neoplasia. Culture and sensitivity analysis yielded a low number of non-enteric gram-negative rods, *Pseudomonas putida*, and *Chryseobacterium indologenes*. The organisms isolated were uniformly resistant to common antibiotics, such as cephalosporins, amoxicillin/clavulanic acid, doxycycline, chloramphenicol, and trimethoprim/sulfa. Based on the culture and sensitivity results, marbofloxacin (50 mg q 24 h) was prescribed for 2 months. The day following the procedure, the client updated that the dog exhibited an immediate remarkable improvement in his level of comfort. At the 2-week recheck, the dog had more energy, allowed a full oral examination, and had a good range of motion of the TMJ. Unfortunately, about 2 weeks after this recheck appointment, the dog exhibited a sudden severe decrease in the energy level and appetite that did not respond to hospitalized supportive care for 24 h and was humanely euthanized.

## Discussion

In this report, we describe our approach to the diagnosis and management of suspected septic arthritis of the TMJ in dogs and exemplify it with reports from our experience with four dogs. Overall, septic arthritis is a rare clinical entity, but when presented, it provides a diagnostic and therapeutic challenge. Affected dogs typically exhibit profound pain and dysfunction necessitating urgent and precise diagnosis in order to identify the correct targeted therapy. In that context, a surgical approach to the TMJ, in order to (1) obtain diagnostic samples for culture and sensitivity, (2) decompress the joint and the associated abscess (if present), (3) remove joint debris, and (4) copiously irrigate the joint with sterile saline to reduce the inflammatory burden, was demonstrated to be beneficial in managing the disease.

A previous study demonstrated osteoarthritis of the TMJ (TMJ-OA) to be the most common disorder of the TMJ in dogs, followed by trauma (22, 23). From the standpoint of biological behavior, osteoarthritis or degenerative joint disease is considered a “low-inflammatory” arthritis (7, 23, 24). We have found that TMJ-OA is symptomatic and is associated with mild to moderate pain or merely discomfort in only ~25% of the cases (22). In contrast, septic arthritis of the TMJ is considered a “high inflammatory” arthritis and is presented with a pronounced pain and dysfunction (i.e., limitation in the range of motion), stiffness, swelling, and potentially the presence of a draining tract. Septic TMJ is typically caused by bacteria, fungi, viruses, or parasites (6, 7, 25, 26).

While it may be difficult to assert the precise initiating cause of septic arthritis of the TMJ, we observed and reported, from a literature point of view, the following four potential scenarios: local dissemination of infection from adjacent anatomical structures, such as the inner ear/tympanic bulla that acts as a continuous focus of infection; trauma (i.e., direct inoculation by a dog bite); penetrating stick injury to the pharyngeal area; or migration of microorganisms from a distant site *via* hematogenous spread (1–3, 18, 19, 27–29). Regardless of the source and cause, once infection of the joint is established, articular destruction ensues and typically progresses rapidly (6, 30). The rapid proliferation of bacteria within the synovial fluid and membrane-induced activation of the inflammatory cascade result in the influx of macrophages, neutrophils, and synoviocytes, as well as in the accumulation of various high levels of pro-inflammatory cytokines and chemotactic molecules (6, 31, 32). In addition, there is the release of proteases and antigen–antibody complex formation within the joint. This profound inflammatory insult on the joint results in catabolic enzymes, causing damage and degradation to the articular surfaces and the disc of the joint (2, 12, 33, 34).

Advanced diagnostic imaging with CT, CBCT, and/or MRI is essential to confirm the clinical suspicion of septic arthritis of the TMJ (6, 11, 14, 15, 22, 35, 36). In that context, contrast-enhanced CT studies have been reported to be the recommended imaging modality in the assessment of septic TMJ arthritis as they allow visualization of the inflamed soft tissues, presence of fluids (i.e., purulent), and permit evaluation of adjacent clinically relevant structures to determine the presence of concurrent myositis and osteomyelitis (11, 17). If the initial diagnosis by CT is not rewarding and the clinical suspicion remains high, MRI is recommended. The MRI displays good resolution in the detection of joint effusion, purulent material accumulation, evaluation of the articular joint surfaces, and reasonable resolution of the articular disc and adjacent structures, such as the cranial nerves V and VII (14, 35). This was proven to be highly beneficial in Case 2 in the present report. Conventional CT and CBCT display better diagnostic performance in identifying osseous structures and diagnosing bone changes (i.e., erosion and subchondral cysts), in identifying spatial locations, and have less interference with metal implants, if present (11, 15, 37).

In order to confirm the clinical and imaging suspicion of a septic process and provide appropriate antimicrobial therapy, it is valuable to approach the joint surgically and obtain several samples of the tissues and fluids for culture and sensitivity and histopathology (1, 6, 13, 38). Given the small size and tight confinement of the TMJ in dogs, attempting fine needle aspiration of the area may result in negative or insufficient findings (39). As noted in this report, laboratory tests, such as complete blood count and serum biochemistry, may be non-rewarding and/or non-specific and should not be used as a sole method to diagnose or rule out septic TMJ arthritis. A blood culture may be useful in identifying causative organism(s) if culture results are negative (40). Finally, given the clinical examples in this study, obtaining culture and sensitivity samples was essential due to the presence of anaerobic or widely resistant bacteria. Only in Case 2, where the owner declined the sampling of the TMJ, an empirical treatment was given using a broad-spectrum antibiotic. In the remaining three cases, empirical treatment was given after surgery and was modified based on the culture and sensitivity findings.

A surgical approach to the TMJ allowed draining of the purulent content of the joint and its surrounding, removing necrotic bone (i.e., sequestrum), debris, and foreign material. Moreover, the surgical approach allowed the irrigation of the joint compartments and the surrounding area to reduce the inflammatory burden (1, 4, 6, 41, 42). Condylectomy should be considered as the last resort and should be reserved for non-responsive osteomyelitis of the condylar process or in cases that develop ankylosis (1, 12, 43). Unfortunately, given the dramatic insult to the joint and the innate inability of cartilage to repair (44), therapy may not result in a long-term positive outcome, and dysfunction in the form of decreased range of motion may remain.

## Conclusion

Septic arthritis of the TMJ is a rare, painful, and morbid form of arthritis in the dog. Understanding the fundamental aspects of this disease and how the joint may become infected is imperative to identify, diagnose, and treat the condition correctly. Diagnosis requires obtaining a good history, a thorough clinical examination, and the application of advanced diagnostic imaging. Furthermore, obtaining diagnostic samples of the joint *via* an open surgical approach proved beneficial in applying the targeted antimicrobial therapy and allowing for a rapid return to normal function. Finally, delayed diagnosis and treatment and non-specific (and ineffective) empirical therapy may result in loss of function, continuous pain, and overall poor outcome.

## Data Availability Statement

The raw data supporting the conclusions of this article will be made available by the authors, without undue reservation.

## Ethics Statement

Ethical review and approval were not required for the animal study because the study is retrospective in nature and included clinical cases, hence, it is exempt from IACUC requirements. The standard written informed consent required for all procedures performed at the William R. Pritchard Veterinary Medical Teaching Hospital of the University of California, Davis was obtained from the owners.

## Author Contributions

BA: study concept and design, provision of study material or cases, manuscript writing, data analysis and interpretation, and review of the manuscript for important intellectual input. NV: provision of the study material, manuscript writing, data analysis and interpretation, and review of the manuscript for important intellectual input. AF: provision of study material or cases, manuscript writing, and review of the manuscript for important intellectual input. FV: provision of study material and review of the manuscript for important intellectual input. All authors contributed to the article and approved the submitted version.

## Conflict of Interest

The authors declare that the research was conducted in the absence of any commercial or financial relationships that could be construed as a potential conflict of interest.
